# Patterns of Transition of Adolescents in an HIV Care Programme in Peri-Urban Cape Town, South Africa: A Photovoice Study

**DOI:** 10.1177/23259582251362908

**Published:** 2025-09-01

**Authors:** Charné Petinger, Talitha Crowley, Brian van Wyk

**Affiliations:** 1 School of Public Health, 56390 University of the Western Cape, Bellville, South Africa; 2 School of Nursing, 56390 University of the Western Cape, Cape Town, South Africa

**Keywords:** Photovoice, participatory research, adolescents, HIV, transition, South Africa, self-management, adherence, engagement in care

## Abstract

Successful transition from paediatric to adult HIV care programme is a critical developmental milestone in the care trajectory of adolescents living with HIV (ALHIV). The transition process involves a shift from a structured, caregiver-supported healthcare model to one that requires independence and self-management. This process should be guided and supportive to ensure continued engagement in care and optimal adherence when ALHIV are transferred. This study utilised photovoice methods to explore the transition experiences of ALHIV in the Cape Town Metropole. Audio-recorded focus group data were transcribed verbatim and subjected to reflexive thematic analysis. Three distinctive patterns of behaviour from ALHIV were identified as themes. Type 1: *socially reliant, dependent adolescent* who heavily relies on family and peer support and struggles with adherence. Type 2: *socially disconnected, hyper-independent adolescent*, who is self-reliant, seeks solitude, and is generally resistant to external support. We configured a third (ideal) type, who is *interdependent* and able to self-manage their chronic condition, but within a supportive health care environment that provides positive healthcare and transition experiences. The findings underscore the need for supportive transition models promoting self-management skills, while facilitating a symbiotic relation with healthcare staff promoting sustained engagement in care well into adulthood. We recommend that adolescent or youth friendly services for ALHIV be expanded to support and monitor the transition process and outcomes in the adult HIV program.

## Plain Language Summary Title

Plain Language Summary

N/A

## Introduction

South Africa has one of the highest burdens of HIV globally, with an estimated 7.8 million people living with HIV in South Africa. Adolescents living with HIV (ALHIV) account for 4.2% of this population.^1^ The roll-out of antiretroviral therapy (ART) in South Africa is marked by a universal test-and-treat approach, which includes the immediate initiation of treatment for all individuals diagnosed with HIV, and the adoption of dolutegravir-based regimens. Despite the advances in ART regimens, which contributed to increased life expectancy, adherence remains a persistent challenge due to stigma, mental health difficulties, and access to healthcare.^2,3^ The transition to adult HIV care further complicates adherence, as it requires adolescents to take on an increased responsibility of their health, while navigating developmental milestones.^4,5^ This transition period is characterised by pervasive challenges, in the form of maintaining adherence to antiretroviral therapy (ART), while navigating less accommodating healthcare systems, and coping with the psychosocial complexities of living with a chronic illness.^6,7^ The successful transition from paediatric to adult HIV programmes is regarded as an important developmental milestone in the care trajectory of ALHIV, as it marks a critical shift in both responsibility of care and individual autonomy.

As of 2023, 320,000 ALHIV in South Africa are registered on ART and are expected to transition by 2030.^8–10^ As they mature into adulthood, ALHIV must shift from a structured, caregiver-led healthcare system (paediatrics), to one requiring greater independence and self-management (adult HIV programs).^11,12^ Biological, social and psychological changes are prevalent during the developmental period of adolescence.^13^ As a result, ALHIV are classified as a priority population, particularly as adolescents are found to have some of the lowest rates of adherence compared to other groups living with HIV.^2,14,15^

Familial structures and peer relationships play a critical role in shaping self-management skills and emotional wellbeing for ALHIV.^16,17^ There remains a paucity in research on the lived experiences of ALHIV who are transitioning or have transitioned to adult HIV care.^18,19^ It is imperative to describe and explore how ALHIV navigate their health, relationships and sense of self during transition to inform the development of tailored interventions to ensure optimal adherence and persistent engagement in care. The current paper reports on a photovoice study that was conducted in a peri-urban setting in the Cape Town Metro of South Africa to explore the transition experiences of ALHIV on ART.

## Methods

### Study Design

This study employed photovoice methodology to explore the transition experiences of ALHIV in the Cape Town Metropole, South Africa. Photovoice is a participatory, visual research method that empowers participants to document and reflect on their lived experiences, often accompanied by narrative explanation and group dialogue.^20,21^ This participatory research method allowed participants to delineate complex experiences and understandings through the use of pictures.^22^ Moreover, the integration of both pictures and direct quotes from participants ensures a thick description of adolescents’ experiences of transition to adult HIV care, while facilitating peer interactions between participants.^22,23^ In the South African context, photovoice methodology has been employed to explore the emotional, social and structural dimensions of living with HIV among adolescents, particularly regarding their healthcare journeys.^21,24^ Photovoice was selected for this study to ensure participants can take control of their own narrative while capturing the layered realities of ALHIV regarding the transition to adult HIV care.

### Study Context

This study took place in a district hospital in the Cape Town Metropole, which provides specialised care to adolescents living with HIV. The hospital is situated in a peri-urban area approximately 40 km from the Cape Town central business district (CBD). Of the 3500 patients who access this facility's outpatient department, approximately 80 adolescents living with HIV (aged 10-19 years) are in care at this facility.^25^ The services provided to ALHIV at this facility include standard HIV services, as well as group sessions facilitated by a healthcare provider to share experiences every two months. This facility provides paediatric and adult HIV care to adolescents. They are transferred to the adult HIV program between the ages of 15–17 years old within the same facility.

### Sampling and Participants

The participants who were included in this study were ALHIV aged 13–19 years on ART and receiving care at the aforementioned healthcare facility. Participants were purposefully sampled after initial identification by staff members in the HIV outpatient department. Therefore, the criteria for inclusion were that participants were disclosed to about their HIV status, on ART, between the ages of 10–19 years old at the time the study took place and were willing to take part in the photovoice project comprising of three sessions and group discussion, as explained by information sheets and consent forms. Despite the inclusion criteria, no 10–12-year-old participants were included in this study, which was largely due to these participants not being fully aware of their HIV status, as indicated by their healthcare provider. Recruitment into the study occurred over 2 weeks. Nineteen participants completed all three photovoice sessions. Table 1 below describes the summary characteristics of the study participants.^26^

**Table 1.** Summary Characteristics of Adolescents Living with HIV (N = 19).

	Sub-category	Total
**Age (in years)**	13–15	6
	16–19	13
**Sex**	Female	9
	Male	10

### Study Procedures

The team involved with recruitment and data collection comprised the first author and a research assistant. Both team members received photovoice training from an experienced photovoice and HIV researcher. The methods used were adapted from US settings and piloted in the Western Cape prior to the commencement of this study.^21,24^ The photovoice projects included three sessions. The first session concerned the recruitment of eligible participants, whereby they were provided with information sheets and consent forms in the language of their choice to explain the purpose of the study. The second session required participants to return signed consent forms, as well as assent and parental consent forms for participants under the age of 18 years. Thereafter, the researcher reiterated the purpose of the study, thoroughly explained the study, and provided smartphones with cameras. In addition, participants were given an instruction sheet which explained that participants are not to take pictures of themselves or others (in accordance with the South African legislation of the protection of personal information) and advised them to prioritise their safety and the safety of their smartphones, as the majority of participants resided in this peri-urban area. Subsequently, participants were instructed to take 3–5 pictures of anything that encapsulates their experience of growing up with HIV, their experiences receiving care at their current healthcare facility, and their feelings about taking more responsibility for their HIV management, including self-management strategies. In the third and final session, participants selected three pictures they discussed them in a group setting and provided captions for each picture as a group. Participants were not financially reimbursed for their participation; however, they received a meal at the final session and were informed afterwards that they could retain the phones they received during the study.

### Data Collection

Data collection for this study was conducted between November – December 2024. As mentioned previously, participants took pictures of their experiences and took part in a group discussion. The group discussions centered around the participants’ pictures, wherein the researcher probed the participants on their pictures and allowed other participants to share their experiences. Pictures were displayed- for all participants in each group to see- through a mobile projector operated by the researchers. Each focus group consisted of 2–4 participants of all genders and lasted between 30 and 90 min. The focus groups were stratified by age, as younger participants (13-16 years) were placed in the same groups, whereas older participants (17-19 years) were placed in different focus groups. Although the study included 19 participants, data saturation was determined as guided by Braun and Clarke's (2006; 2019) notion of theoretical sufficiency after the 17^th^ participant, and an additional two participants were included to confirm that sufficient information power had been achieved to address the research aim. The focus group sessions were audio recorded and transcribed verbatim by the first author.

### Data Analysis

Data were analysed thematically using reflexive thematic analysis following the approach outlined by Braun and Clarke.^27,28^ After the transcripts were uploaded to Atlas.Ti version 25, the pictures were embedded at the corresponding timestamps where they were discussed in the transcripts. As outlined by photovoice methodology, the data consisted of two interlinked forms, participant-produced pictures, and focus group discussion transcripts. As mentioned previously, the participants’ images were embedded into the transcript to allow for the contextual interpretation of the visual content within the narrative of the group dialogue. While the visual content was not coded in isolation, it was discussed and interpreted by the participants, and reported as such, to ensure that the assigned meaning remained participant-led. Reflexive thematic analysis was beneficial to this study as it enabled a flexible yet rigorous framework, allowing for an in-depth exploration into the transition experiences of ALHIV. This was done through inductive coding to allow themes to emerge organically concurrent to the researcher interpretation, rather than to impose a pre-existing framework. The codes were developed iteratively, with regular memo writing during team discussions and initial coding to document analytic decisions, shifts in interpretations and emerging conceptual linkages. These discussions and reflections reported on researcher subjectivity and positionality, which is consistent with the reflexive ethos of this analytic approach.

### Rigour

Trustworthiness was maintained through the concepts of Lincoln and Guba.^29^ This included credibility, dependability, confirmability, and transferability.^30,31^ Credibility was maintained through providing thick, rich descriptions of the data. Dependability was maintained through following the procedures mentioned above for data collection. Confirmability was maintained through the first author keeping a reflective journal throughout the process of recruitment and data collection. Confirmability was further maintained through regularly debriefing with the research team, which took place during recruitment of participants, and after each photovoice session. Transferability was maintained through rich descriptions of both study context and participants, which can be transferred to future research endeavours.

### Ethics Considerations

Ethics clearance was obtained through the first author's registered university, as this photovoice study forms part of the first author's doctoral research (BM23/6/5). Further, permission to access the selected facility was granted by the Provincial Health Research Committee (WC_202308_043). Through the provision of information sheets and consent forms to participants, they were made aware of their rights as research participants. This included being fully informed about what their participation would entail, that they were free to withdraw from the study at any time without negative consequences to them, and that all personal and identifying information would be made anonymous and kept confidential. The use of pseudonyms ensured that anonymity and confidentiality were maintained. The collected data was stored in a secure, password-protected folder with the first author and her supervisors having sole access.

## Results

### Overview of Themes

From the reflexive thematic analysis of the data, two interpretative “archetypes” of participants were constructed, which served as an organising framework for the thematic narrative and their experiences within the healthcare facility. While Braun and Clarke's (2023) approach provided the structural foundation for theme development, the identification of these overarching archetypes occurred in the later, more interpretative phases of analysis, as deeper interpretive patterns began to coalesce in the data. Consistent with the interpretive nature of reflexive thematic analysis, participants were not quantified within the archetypes. The first archetype–the socially reliant, dependent adolescent–describes the participant who is not ready to transition or not ready to manage their care. The second archetype–the socially disconnected, hyper-independent adolescent–characterises the participant who relies on themselves for their health and emotional sustenance. In the third theme (interdependent), participants delineate the care they receive at the facility. It is an experience that is shared across both “archetypes” of participants and denotes the support necessary for developing sustained emotional connection while enforcing HIV and treatment literacy and capacity building. The themes are described in Table 2 below.

**Table 2.** Thematic Analysis.

Archetypes	Description	Typical Characteristics
**Type 1: The socially-reliant, dependent adolescent**	Struggles with adherence and transitioning	Reasons of disengagement from care
		(Negative) feelings about growing up
		Feelings of loneliness and isolation
		Negative feelings about living with HIV
		Initial fear of healthcare facilities
		Lack of HIV literacy in paediatric care
		We don't want to wait long at the clinic
	Dependence on social support and external guidance to cope	Feeling liberated through social connections
		Feeling understood through friendships
		Relying on faith for strength and comfort
		My family supports me so I can remain adherent
		(Material) Things that make me feel safe and secure
		Healthcare worker assistance to ensure that I am adherent
**Type 2: The socially disconnected, hyper-independent adolescent**	Self-sufficiency and independence	Creative exercises as a form of coping
		Gaining strength through independence
		Gatekeeping personal issues
		Learning from my own mistakes
		Learning from others’ mistakes
		Seeking solitude
		Intentional self-regulating practices
		Deliberate social disconnection
	Internalised responsibility to remain healthy and adherent	Acceptance of living with HIV
		Acknowledging the importance of my treatment
		Knowledge and understanding comes with age
		Remaining healthy is embedded in me
		Strategies I take to remember to take my treatment
		Resilience through self-care
**Type 3: The “ideal” Interdependent and supported adolescent**	Positive experiences with health services	Compassion and care from healthcare workers
		Satisfaction with health services
	Developing knowledge and skills post-transition	Increased knowledge about HIV after I transition
		Positive experiences of seamlessly transitioning to adult care
		Post-transition practices to remain adherent
		Treatment knowledge and literacy
		Understanding the process of transitioning

### Type 1: The Socially Reliant, Dependent Adolescent

The first theme, or archetype of adolescent, describes the adolescent who may be struggling with accepting their HIV status, managing their care and relying heavily on their social support structure for emotional sustenance. Participants discuss how, when they were disclosed to regarding their HIV status, they had difficulty in internalising and believing it:

*I took them seriously, but I like… didn’t believe them. (P2, Female, 16)*

Participants further explain that acceptance of their HIV status is something that they also struggle with.

*So, I think the title and the whole concept of why it's off is because it's a constant reminder of my torture and the things that I didn't deal with because I haven't gotten over the fact that I'm in the hospital. And if I don't even say it loud in front of people that, yeah, it's difficult to adjust and just live with it and be like, oh, wait, this is my life. I can't just say this is my life. (P16, Female, 14)*

Participants fitting this archetype further show difficulty in remaining adherent and engaged in care, which is underscored by their negative feelings about living with HIV and negative experiences at healthcare facilities. This is further exacerbated by fear of healthcare facilities, as it may represent a place of illness and mortality:

*At first, I was scared, anxious, and just not comfortable with the idea of being at the hospital alone. I like being at the hospital, but I don't like being a patient. Or just attending things. If it's being at the hospital, let it be about my career. (P16, Female, 14)*

Moreover, participants do not prefer coming to the clinic, particularly as a result of the long waiting times:

*It feels so long, because I am sitting the whole time. (P13, Male, 14)*

When posed with the idea of transitioning to adult care, which represents participants having to take care of themselves, participants describe a sense of apprehension:

*It's too much to handle. (P8, Female, 15)*

*I'm not ready for the world. I don't feel like I'm ready. (P9, Female, 19)*

Furthermore, adulthood represents a challenge for the participants, even for those who have already transitioned to adult care:

*From [paediatrics] to adults, I feel like that was very challenging. Like, the reason I took that [picture of my rugby certificates] is because you have a lot of challenges in a rugby game… it is challenging. (P14, Female, 16)*

Participants within this archetype are ones who heavily rely on external sources for guidance and support. Participants explain that they rely on their religion to cope, finding strength and comfort in their faith:

*So that is basically my comfort zone. That is where I lash out. Tell everything. Whatever happens. Even though He knows what happened throughout my day. I just feel like telling Him makes it even better. Because He will just rectify the situation without me knowing. And then by the day, when I wake up, it's gone. He is rectifying it. (P17, Female, 17)*

**Figure 1.**
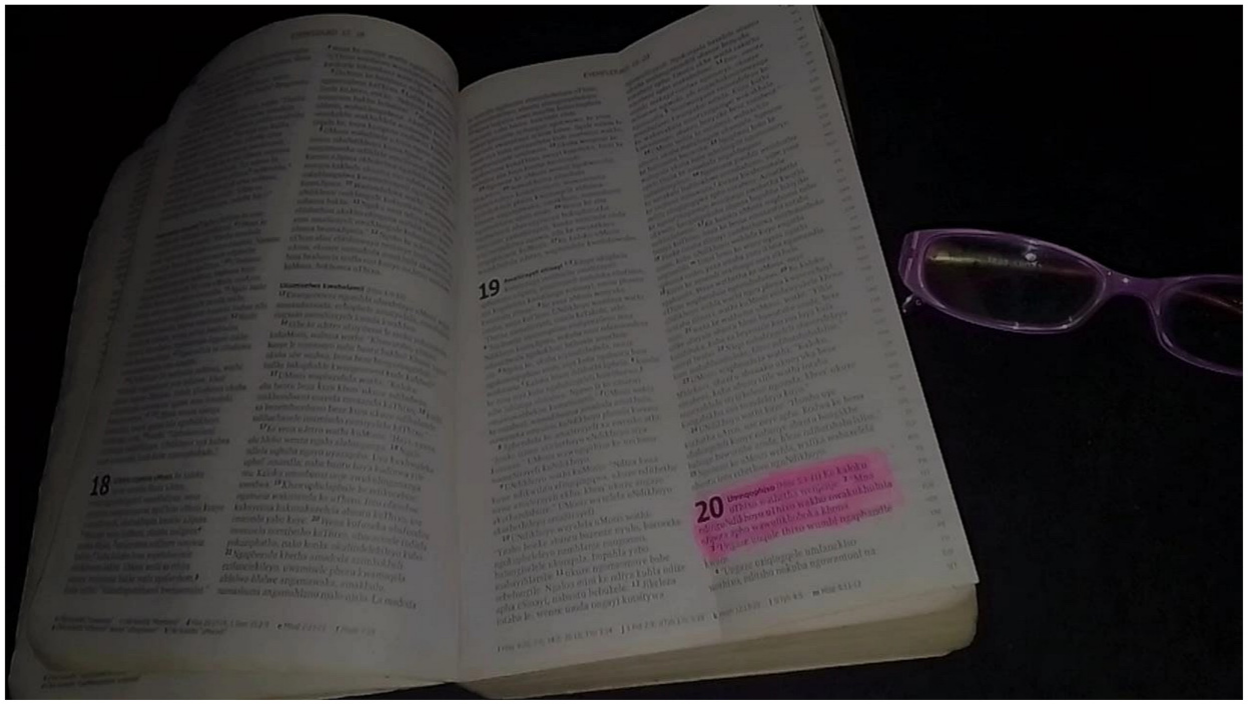
My source of strength.

Participant 17 strengthens this through a picture of the Bible:Figure 1.

*I think that's what kept me going. Also, because I read the Bible and I was like, oh, that's worth it. He helped me. (P16, Female, 14)*

Participants further bond with their family members through religion, particularly in times of difficulty:

*Then we pray. We ask God to protect us. (P5, Male, 13)*

Their dependence is also seen through relying on their caregivers and healthcare providers to make decisions about their health and play a major role in ensuring they remain adherent. Participant 15 explains that even when she is struggling to remain adherent, her family supports her:

*But everyone supports me, even when I drink my pills, they encourage me too. Sometimes, they will force me to drink them if I don’t want to. (P15, Female, 19)*

**Figure 2.**
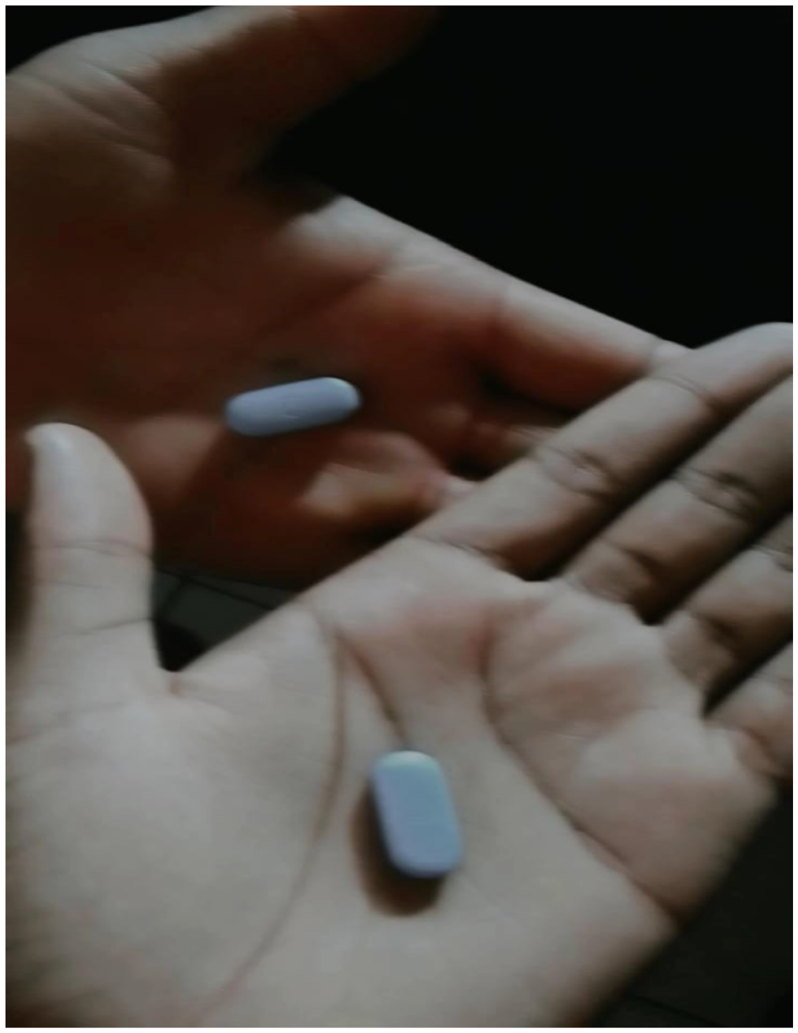
My pills, my life.

In instances where there are multiple people living with HIV in the family, participants explain how they take their medication together, remind one another, and rely on each other to share difficulties they may be having. Participant 8 shares a picture captioned, “My pills, my life”, to strengthen this:Figure 2.

The participants falling within this archetype all explain how they depend on their families, parents, or healthcare providers to help them with their healthcare management. Participant 11 explains:

*Those two people, my parents, tell me what time to drink my medicine on time. (P11, Male, 16)*

Moreover, the support they receive from their caregivers is on their terms, where the participants can assert their need for more or less support. Subsequently, the participants derive their emotional strength through social connections, particularly friendships. They further explain how their friendships serve as a liberation from living with HIV. Participant 2 explains:

*Like, it brings me more freedom to be with my friends rather. (P2, Female, 16)*

She explains that her friends understand her more than her family, despite them not being aware of her HIV status, and this sentiment is echoed by other participants.

*They will understand if I am going through something… they will say this and that. (P2, Female, 16)*

Participants derive emotional sustenance from social connections. Often, they explain that living with HIV is difficult, a challenge, and through their friends and family, the negative feelings that occur are alleviated.

*People don't understand that. The little things can make big differences. So that's why I'm like, even the little conversations… And it actually feels good. It's a good thing. (P17, Female, 17)*

### Type 2: The Socially Disconnected, Hyper-Independent Adolescent

This theme describes the archetype of participants who demonstrate self-sufficiency and independence of taking care of themselves, and an internalised responsibility to remain healthy and adherent.

The participants described the different ways in which they take care of themselves and how they are self-sufficient. Oftentimes, this is through relying on internal coping strategies rather than external sources of support.

*And I realise I have to push myself, whether I like it or not. You go through the pain. (P7, Male, 18)*

When asked how they deal with difficult situations, participants explain how they navigate it on their own, albeit with reluctance. Further, seeking solitude, or choosing to be alone, is a way in which the participants can navigate their emotions.

*If I want to clear my head, I sit by the beach and then I take pictures of the sky or a pretty view. (P1, Male 19)*

Moreover, participants explain how remaining on their own and not trusting other people with their well-being is preferable to them. Participant 1 reflects on this, explaining how he feels that he cannot rely on others for support:

*There's gonna come a time where I’d have to think for myself that no, I have to be independent. I must be able to depend on myself. (P1, Male, 19)*

Participants also explain that they can mature faster and learn how to navigate difficult situations if they only rely on themselves. As a result of this, participants have decided to gatekeep their personal challenges. That is, whether it is regarding their HIV status or any other stumbling block, they would not tell anyone, including their family.

*I've never told anyone. It's not their business. (P4, Male 18)*

This also carries over to the relationships with their close family or parents, as participants explain that they simply do not speak to anyone when they are having difficulties.

*I think my blocking and deleting started when I was in grade 9 already. Like, I had grown up. Because I would block my dad also. I would block my sister. I would block everybody. (P17, Female, 17)*

Participants expressed that it is important for them to take care of their health, as they have often seen with others and themselves, the negative consequences of not remaining adherent.

*It was important for me to remember because my mother almost died for not taking her medicine. (P9, Female, 19)*

As seen with Participant 9 above, other people's mistakes represent an intrinsic motivation for them to remain healthy. The participants further discuss how they can acknowledge that taking their treatment is important to them, and oftentimes, they can be hard on themselves if they do not manage it effectively.

*Because it's almost like you have a child, you have to care for it. Now I have pills I have to take, you know. So, like, it feels like a big responsibility because, like, if I forget sometimes, I’m disappointed it myself, but then I think I am fine. (P14, Female, 16)*

The participants also note that they learn from their own mistakes as well and can take accountability for them. Particularly when they do not take their treatment as regularly as they should. Participant 15 expands on this:

*Back when I didn’t take my pills regularly, then I couldn’t move my legs for the day. Or I can’t do anything. But that I know was obviously my own fault. I blame no one. (P15, Female, 19)*

As a result of participants gaining strength through independence, they maintain a positive outlook on transitioning to adult care.

*So, the message is… It's kind of like free. You can do basically what you want. You can come to the clinic. Take your meds anytime you want without anyone checking If you took your meds. All that stuff and being on your case. On your own. You can make your own decisions (P10, Male, 19)*

**Figure 3.**
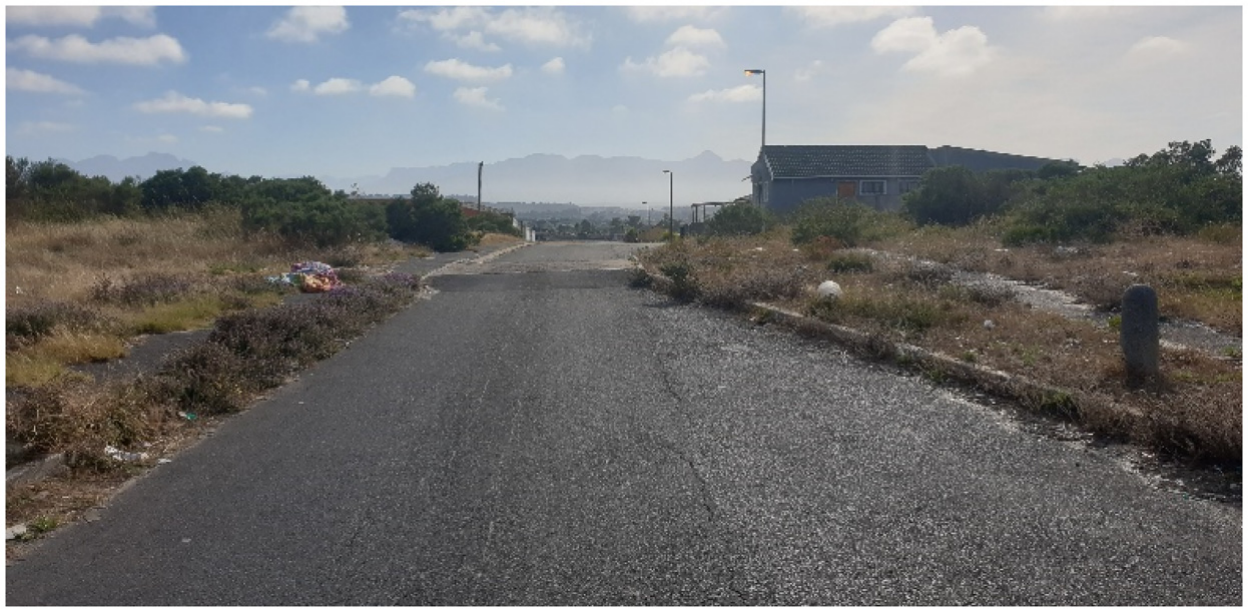
No pain, no gain.

Participant 7 echoes this through sharing a picture of a road captioned “no pain, no gain”, explaining that he thinks of maturing as the long road he takes coming home from school. He equates growing up to being exhausted, but relying on himself to push himself to get to his destination Figure 3.

**Figure 4.**
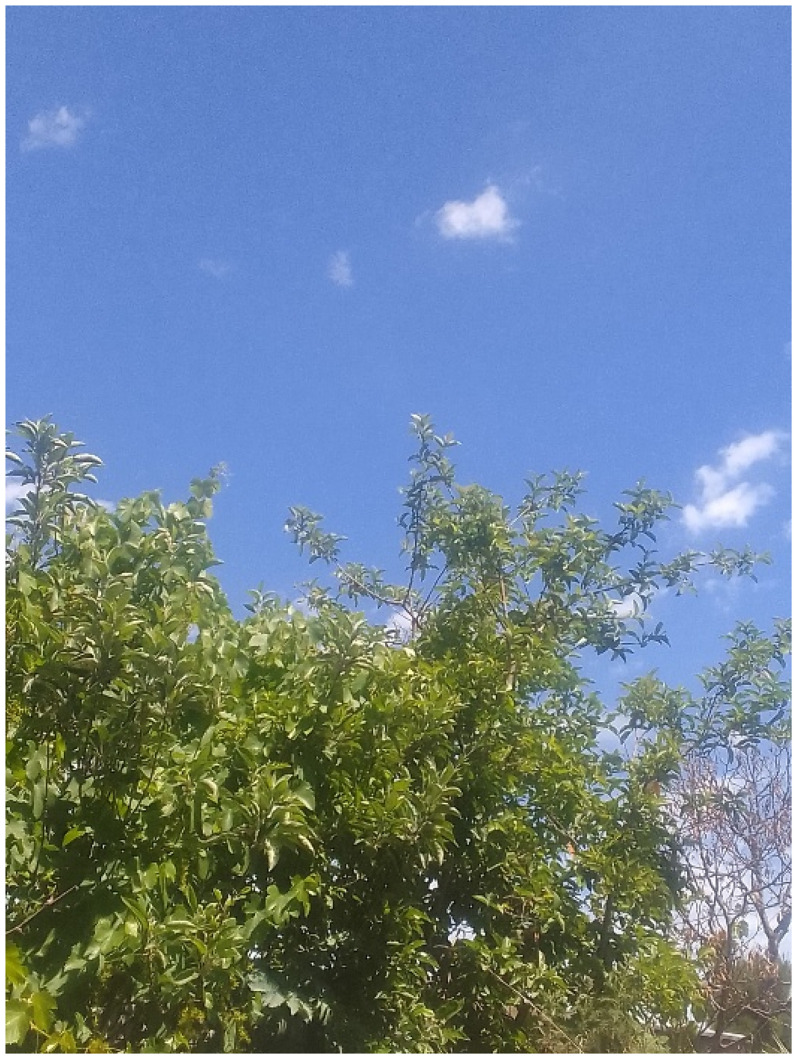
Transitions in nature.

Participants who have already transitioned to adult care explained it as a positive experience. While they were not told that they would transition and how to take care of themselves, it helped that they transitioned within the same facility. Participant 9 explains this through a picture of a tree, to show that the transition was a natural process for her Figure 4.

While the participants demonstrated a sense of independence and healthy behaviours, they also showed a mistrust of other people, which emerged from fears of being judged or treated badly.

*And I'm also secretive. I do not tell you. Because sometimes they just don't get it. Yeah. And they never will. (P17, Female, 17)*

Thus, participants maintain a fixed perspective on how other people may perceive them, especially regarding their chronic illness. Participant 13 strengthens this through fearing that if she discloses to someone, they will mistreat her. As a result, participants have come up with creative ways to explain why they take medication, often through misdirection or lying about the reason for the pills.

*Other people stay by me so those people know nothing about this. So, for them it's weird to see me drink pills. So, then I’d have to tell them, “You guys know I’m not right in the head, I have to drink these pills”. (P15, Female, 19)*

Their creativity is transferred to finding ways in which they can supplement the lack of support and care they receive:

*Yeah, I like to dress up nicely, cause before I couldn’t… but fashion is a part of my life. (P1, Male 19)*

Art is a way participants can make sense of their feelings, self-soothe, and escape from their challenges.

*Uh mainly just my phone. I start to draw. Listen to some music. Anything I, anything I don’t do in daytime (P7, Male, 18).*

*Because the pictures are not about me. So it's easier to make sense with what I'm feeling or how I'm feeling at the same time (P16, Female, 14)*

Other participants take part in different sports to ease tension and release stress:

*Sports takes me away from the stress. Especially during exams also. Exercise helps. (P3, Female, 18)*

Finding these creative ways is how participants can maintain a positive outlook in life. Participant 17 explains this:

*So the thing is, even how dark you are hold on to the little light you have because it can be a bigger spark. (P17, Female, 17)*

Participant also find creative ways to remain adherent, as they explain how taking their treatment is ingrained in them. Participant 7 explains how water reminds him to take his treatment:

*Because, it reminds me of the taste of water. It was like something, like when I drink water, it's almost like certain thing I have in my tongue. (P7, Male, 18)*

Participants, therefore, find things to remind them to take their daily treatment. Participant 15 explains that when she waters her plant, it not only represents her taking care of herself, but serves as a physical reminder that it is time to take her medication. Participants describe their acceptance of their chronic illness and an acknowledgement that they are responsible for their own health:

*I feel normal as someone with HIV. Because it becomes normal. I do certain things over and over, like coming to the clinic, I take my medication, I come to the hospital. (P13, Male, 14)*

Moreover, participants explain that growing up with a chronic illness is something that they have to do on their own.

*You know, but then I think that this is something I have to do for myself, it's not for them [my family] that I have to do it. Like it's for myself and my own health, so I just have to. (P14, Female, 16)*

Participants have come to this realisation by themselves, and this further demonstrates their unwavering commitment to being independent and self-reliant.

*Because I realised that either way, I'm gonna have to face the fire alone. Because this is my journey. (P17, Female, 17)*

Therefore, the participants in this archetype demonstrate a strong sense of agency and resilience, particularly relating to their health-related challenges, although their social disconnection drives them further inward.

### Type 3: Interdependent

Despite the absence of adolescent-specific dedicated services, participants have positive experiences with the facility and their healthcare workers, particularly the participants who have transitioned to adult services at the same facility. This theme, therefore, describes positive experiences with the delivery of health services and the care they receive from the healthcare providers.

*There's no hassle [at the facility], not a thing. Nothing. (P1, Male, 19)*

Participants explain that because they are in clubs, meaning that their medication is already pre-packed for pick-up, it is efficient. The facility also provides a “buddy system” where family members can pick up their medication for them.

*I can send people to come fetch my medication and bring it to me. (P5, Male, 13)*

Regardless of the efficiency of the facility, participants find it helpful to physically come to the facility.

*I feel like good about coming here. It helps me a lot. (P8, Female, 15)*

Through showing a picture of the sky, one participant explains that she feels calm when she is at the facility:

*This is how I feel when I sit at the hospital. I feel calm. The sky brings the calmness. (P9, Female, 19)*

**Figure 5.**
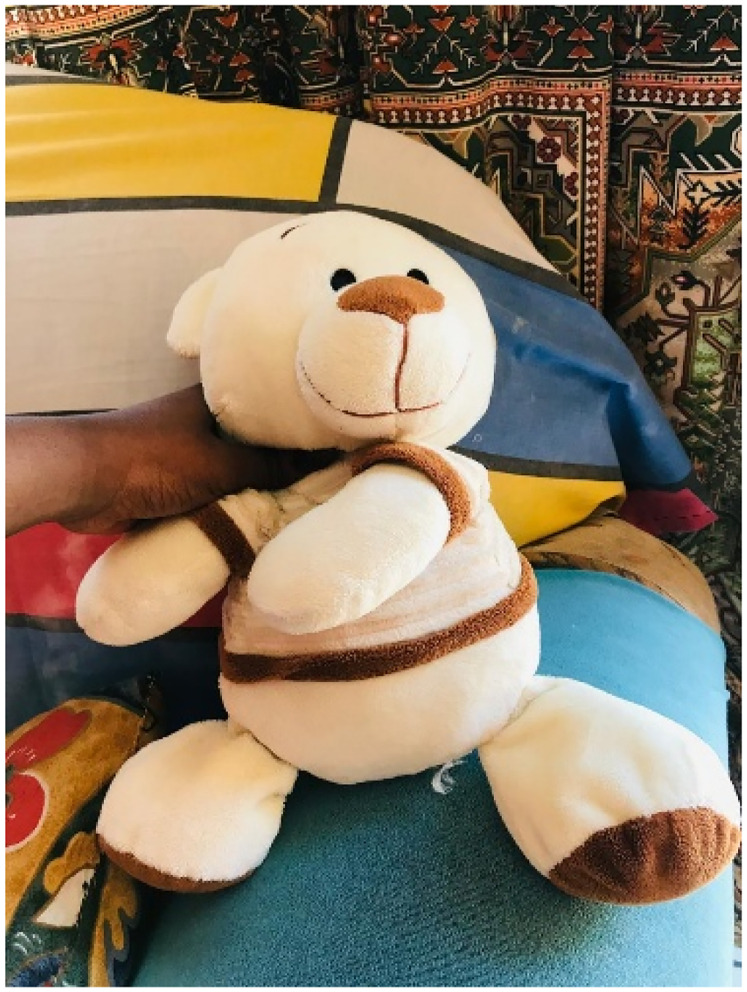
Comfort.

Moreover, the participants are in agreement that the staff at the facility aids in them experiencing the facility as pleasant and welcoming. The participant shows this through a picture of a teddy: Figure 5

She explains:

*So the teddy bear, um it explains the or reminds me of the like comfort that I felt from the doctors. (P3, Female, 18)*

Participant 6 strengthens this finding, as they feel a sense of compassion and care from the staff:

*Nah, it feels better to me, because it shows us that the people care about us, and we are important to the hospital, and they just keep us safe and healthy. (P6, Male, 19)*

Furthermore, participants have positive experiences after transitioning to adult care. They explain the process as natural, yet a change of pace, being that it did not feel as if they were in a serious hospital set-up.

*Honestly, it was more like… Like a different feel to it. Like it was more energetic, more casual and more like… Since I saw her the most, I felt most comfortable with her. That's how I would describe it. (P7, Male, 18)*

Because of their comfort with the facility staff, participants did not note the change in primary healthcare provider in the physical transition:

*No, everyone knows me already so, it's [transitioning to adult care] nothing big. (P15, Female, 19)*

The transition has also been explained thoroughly to some participants, as they explained that the healthcare providers have explained what it entailed.

*The fact is that they explain everything nicely to make sure I understand it well. (P1, Male, 19)*

However, some participants noted that it was initially challenging being in adult care, but they became more comfortable as they returned for the following scheduled visits:

*It was challenging the first time, but now I just kind of like, okay. That's the norm. (P3, Female, 18)*

Transitioning to adult care also allowed the participants to learn more about living with HIV. For some, disclosure of their HIV status happened as they were in the process of being transitioned to adult care.

Participants learned creative ways to remain adherent in adult care from their provider, as well as how to lead healthy lives, such as by eating balanced meals. Participant 11 furthers this, explaining that he did not know much about how his health is being managed until he transitioned to adult care:

*So like, this is like before when I first started coming to her [the adult doctor]. So…* *Like when I was a child, I didn't really know like much about what was going on. I was kind of like…* *Kept out of like knowing things. (P11, Male, 16)*

Therefore, a supportive healthcare provider or facility assists the participants in actively engaging with their care and experiencing a sense of support. We find that a supportive, capacity-building healthcare environment can support both archetypes of ALHIV, as identified previously, in order to facilitate interdependence for the adolescents.

## Discussion

The findings from this study delineate two archetypes of adolescents, as well as structural factors that could assist in the development of an “ideal” type of ALHIV. The first archetype we describe is the socially-reliant, dependent adolescent. Participants describe how they rely on family, peers and healthcare workers for emotional sustenance and treatment adherence. It is evident that adolescents living with chronic conditions such as HIV require additional support, particularly concerning their treatment and their psychosocial well-being.^32^ Thus, the findings that emerged from this theme highlight ALHIV's needs for additional support, and the different ways in which support can manifest. However, while the support may be protective, it can also be limiting, as it may inhibit the development of self-management skills and independent engagement in care.

Reliance on others for coping and decision-making can delay the development of self-acceptance, which may reinforce negative self-perceptions as well as limit opportunities to critically engage with their identity as a person living with a chronic condition.^33,34^ Subsequently, ALHIV within this archetype may face difficulty in transition readiness as a heightened dependence on external support may delay the acquisition of key skills such as self-management, health-related decision-making and independence.^33,35^ The transition to adult HIV care is characterised by the expectation that ALHIV has a higher degree of independence.^19,36,37^ As such, without optimal preparation and readiness to transition, ALHIV may struggle to navigate treatment adherence and decision-making, as well as a less structured and more autonomous adult care programme.^5,38^ In such cases, it may be beneficial to incorporate frameworks such as the social-ecological model of adolescent and young adult readiness to transition (SMART) framework.^39^ This tool can help healthcare providers, caregivers and ALHIV to ensure that they are adequately prepared, emotionally equipped and practically skilled to manage their HIV care as they transition into adult care.^10,39^ It is commonly applied in existing literature to assess transition readiness to assess factors such as health literacy and self-efficacy, across individual, interpersonal, and structural levels to inform the development of targeted, adolescent-specific interventions.^39^

The second archetype refers to the hyper-independent, socially-disconnected adolescent. These participants are characterised by their self-sufficiency, emotional gatekeeping and preference to managing their care in isolation. Their independence appeared to foster a heightened sense of responsibility and need for control over their health, which aligns with core competencies in frameworks such as SMART. This includes self-management skills, decision-making, and autonomy.^39^ However, their hyper-independence was often concurrent with a deliberate withdrawal from social support systems. Their autonomy may support short-term health outcomes, like adhering to their treatment, but the lack of meaningful psychosocial support may limit their access during periods of crisis.^40^ It is evident that a social disconnection may increase their vulnerability to isolation, internalised stigma, and emotional burden, which in turn may compromise sustained retention in care and mental health and wellbeing.^41,42^ This archetype may demonstrate a sense of transition readiness; their limited engagement with psychosocial relationships highlights a critical gap in holistic care. The role of interpersonal support has been found to sustain emotional resilience and continuity in treatment for ALHIV.

The ideal archetype of adolescent is one where they are equally reliant on social support systems and their independence. Rather than presenting one archetype as an ideal form adolescent functioning, we propose interdependence as a balance and context-sensitive goal, which supports adolescents to develop both self-management skills and meaningful connections are created. Interdependence in healthcare refers to a balanced state in which ALHIV possess the skills to manage their health independently while recognising and utilising supportive relationships.^43^ This concept is particularly helpful in the context of ALHIV, as it supports both self-efficacy and connectedness and belonging.^43^ Fostering interdependence for ALHIV will ensure adherence and engagement in care, as well as ensuring emotional resilience and social support.

The role of the healthcare system and its providers is emphasised in the findings of this study, particularly in shaping transition experiences for ALHIV. Participants explained that they had positive transition experiences, particularly noting the transition as “*natural*” and “*well-explained*”. This reinforces the importance of respectful, non-stigmatizing care, and consistent support from healthcare workers can facilitate adherence, agency, and engagement in care.^19,44,45^ Furthermore, participants explained how their interactions with their healthcare providers aided them in gaining knowledge, confidence and a sense of ownership over their treatment. Existing literature recognises that ALHIV often have a limited understanding of their condition, wherein healthcare providers should play a pivotal role in shaping understanding and acceptance of their HIV status through effective communication and supportive relationships.^34,35,46^

The integration of healthcare practices that foster agency, resilience and reflective self-management into routine care may allow for long-term engagement in care and optimal transition to adult HIV services.^16,46^ This allows adolescents to internalise responsibility while remaining embedded in their healthcare systems. Adolescent-friendly services and supportive transition facilities should incorporate respectful communication, youth-led engagement and spaces where peer support is illuminated.^25,46^ Furthermore, it is found that peer-led models of care and family involvement serve as key sources of emotional resilience, which reinforces the idea that the successful transition to adult care does not require complete independence, but rather a well-supported form of autonomy that is embedded within trusted psychosocial relationships.^41,46^ This study further delineated different archetypes existent within ALHIV, and through developing and identifying these archetypes, it may be beneficial in the clinical setting to tailor support to ALHIV who are transitioning to adult HIV care. However, while these findings offer important insights, they must be interpreted within the context of the study's methodological limitations.

While the included sample can be considered a small sample size, it is consistent with typical sample sizes reported in photovoice studies involving ALHIV.^47^ As highlighted in their review, Mayman & van Wyk,^47^ photovoice studies commonly involved small, targeted groups, with sample sizes often ranging between 8–20 participants. The current study falls well within the expected and methodologically acceptable range for participatory research. Data saturation has been achieved as this study population can be considered homogenous as the inclusion criteria for this study is limited, and took place in a setting that has little variation in socioeconomic status as well as culture. Despite the homogeneity which can be considered a limitation in terms of broader applicability, it enhanced the depth and cohesion of the insights of this study. Richness of photovoice data often allows for profound and layered understands of participants’ lived experiences, particularly within small samples.^22,48^

## Conclusion

The findings of this study underscore the need to expand adolescent-friendly health services to include guided and supportive transition ‘protocols’ that would facilitate interdependence in adolescents who are sufficiently educated and aware for self-management, while equally astute and confident to access the health care system and available psychosocial support services when the need arise. We note the inadequacy of health policy and operational guidelines in South Africa for supporting ALHIV in transitioning to adult care; and recommend further development in adolescent and youth-friendly health policies globally.^49^ Comprehensive health policies for adolescent-friendly service should include monitoring systems to report on treatment outcomes post transition as well as ongoing training for healthcare providers to remain adolescent-sensitive. Future research should be grounded in implementation science to ensure that the delivery of adolescent-friendly services include supportive transition, and is sustainable and maintained beyond the research study.^50^ In addition, increasing emphasis should be placed on youth-driven solutions to ensure high acceptability of service delivery models to ALHIV.
